# The evolution of ancient healing practices: From shamanism to Hippocratic medicine: A review: Retraction

**DOI:** 10.1097/MD.0000000000047606

**Published:** 2026-01-23

**Authors:** Chukwuka Elendu

The article “The evolution of ancient healing practices: From shamanism to Hippocratic medicine: A review”,^[1]^ which appeared in Volume 103, Issue 28 of *Medicine®* is being retracted after a concerned reader alerted the Editorial Office of multiple references that do not substantiate the claims they are meant to support. The Publisher investigated and confirmed the author’s submitted reference list contained multiple inappropriate and/or irrelevant references. Additionally, there are concerns that the author appears to have used a Generative AI source in the writing process of the review without any disclosure, which is a breach of journal policy and publication ethics. As a result, the Publisher no longer has confidence in the integrity of the content.
